# Drug treatment of malaria infections can reduce levels of protection transferred to offspring via maternal immunity

**DOI:** 10.1098/rspb.2011.1563

**Published:** 2012-02-22

**Authors:** Vincent Staszewski, Sarah E. Reece, Aidan J. O'Donnell, Emma J. A. Cunningham

**Affiliations:** 1 Centre for Infection Immunity and Evolution, University of Edinburgh, Edinburgh EH9 3JT, UK; 2 Institute of Evolutionary Biology, School of Biological Sciences, University of Edinburgh, Edinburgh EH9 3JT, UK

**Keywords:** passive immunity, neonatal immunology, immunoglobulins, anti-malarial drugs, *Plasmodium chabaudi*, maternal effects

## Abstract

Maternally transferred immunity can have a fundamental effect on the ability of offspring to deal with infection. However, levels of antibodies in adults can vary both quantitatively and qualitatively between individuals and during the course of infection. How infection dynamics and their modification by drug treatment might affect the protection transferred to offspring remains poorly understood. Using the rodent malaria parasite *Plasmodium chabaudi,* we demonstrate that curing dams part way through infection prior to pregnancy can alter their immune response, with major consequences for offspring health and survival. In untreated maternal infections, maternally transferred protection suppressed parasitaemia and reduced pup mortality by 75 per cent compared with pups from naïve dams. However, when dams were treated with anti-malarial drugs, pups received fewer maternal antibodies, parasitaemia was only marginally suppressed, and mortality risk was 25 per cent higher than for pups from dams with full infections. We observed the same qualitative patterns across three different host strains and two parasite genotypes. This study reveals the role that within-host infection dynamics play in the fitness consequences of maternally transferred immunity. Furthermore, it highlights a potential trade-off between the health of mothers and offspring suggesting that anti-parasite treatment may significantly affect the outcome of infection in newborns.

## 1. Introduction

Maternally transferred immunity constitutes an epigenetic mechanism by which mothers transmit their history of pathogen exposure to protect offspring during the development of their own immune system [1]. Maternally transferred immunity is, therefore, an important source of phenotypic variation [2]. It can affect the expression of traits in subsequent host generations and hence underlie variation in resistance to parasites [3]. However, levels of antibodies in adults can vary both quantitatively and qualitatively over the course of infection [4]. To date, few studies have investigated how the factors affecting the dynamics of maternal infection and the resulting immune response might affect the strength and the specificity of protection transferred to offspring. More broadly, variation in the dynamics of maternal infection is a novel experimental framework to study trans-generational responses to environmental heterogeneity and the maintenance of inter-individual variability. As such, maternally transferred immunity can influence how organisms are shaped by the interactions between genetic and ecological factors [3,5]. However, these two factors—the dynamics of infection and host genetics—have largely been overlooked in studies of maternally transferred immunity.

There is nevertheless good reason to hypothesize that the dynamics of maternal immunity may vary over time. Theoretical [6,7], experimental [4,8] and epidemiological [9] studies show that the duration of infection in adults influences the amount and the diversity of antibodies produced by the immune response, thereby affecting the level of protection that could potentially be transferred. This is because the length of infection determines the range, duration and amount of antigen expression by parasites [4,10]. In addition to host and parasite characteristics (such as parasite virulence, concurrent and previous infections, infectious doses, age, sex and condition of hosts), antiparasitic drug treatment can influence infection duration. Consequently, by decreasing the duration of expression, or the range and amount of antigens expressed by parasites, anti-parasite drugs may negatively affect the effectiveness of an immune response [11]. Alternatively, it has also been hypothesized that treatment against parasites could enhance immune responses by altering the different parasite proteins accessible to the host [12,13] and result in a stronger or more directed response. The effect of drug treatment on the acquisition of immunity is particularly important to consider for individuals living in endemic areas, where re-infection is likely to occur and anti-parasite drug treatment is widely used. Furthermore, in endemic areas, offspring are likely to be exposed to the same pathogens as their parents and at an age where their immune system is not fully developed [14]. They consequently rely on the repertoire of antibodies transferred by their mothers to confer passive protection. There may, therefore, be consequences of maternal anti-parasite treatments for the transfer of passive immunity to offspring.

Rodent malaria is an excellent model system to test how infection dynamics and host and parasite genetics interact to shape the protection provided by maternally transferred immunity. Different lines of inbred mice display different patterns of immune responses [10,15], a bank of genetically characterized parasite genotypes are available and infection dynamics can be manipulated using anti-malarial drugs to curtail the duration of the infection [16]*.* Previous studies of maternally transferred immunity to rodent malaria have established that varying levels of resistance to malaria infection could be transferred to offspring by antibodies in milk [17–19]. However, while there are many studies on the implications of malaria contracted by the mother during pregnancy [20], very little is known about factors influencing immune responses of the mothers and transferred immunity, especially when mothers are exposed before pregnancy. Moreover, in the few studies there are, infections in dams were either self resolving or manipulated to boost acquired immunity [21], e.g. up to three consecutive short drug-treated infections. It is, therefore, unclear how the dynamics of maternal infection, standard treatment protocols and associated immune responses may affect maternally transferred immunity. Here, we examine, in three different mouse strains, the consequences of drug treatment for the transfer of maternal antibodies and the health (growth rate, parasitaemia and anaemia) and ultimate survival of offspring. This allowed us to test whether pups born to dams that experienced malaria infection were better protected than pups from naïve dams and whether the maternal transfer of protection is substantially decreased when drugs are used to cure infections in dams and consequently alter the infection dynamics.

## 2. Methods

We experimentally tested whether the dynamics of maternal infection influenced the protection conferred by maternally transferred immunity. For three different mouse strains, we infected dams with malaria and bred from them. Dams were either drug-treated three weeks post-infection, untreated with the infection allowed to run its full course or sham-infected as a control. Their pups were infected with one of two parasite genotypes that differ in virulence, or sham-infected. We measured the amount of maternally transferred antibodies and the morbidity and mortality experienced by pups.

### (a) Parasites and hosts

We used three different strains of mice: two strains of inbred mice C57Bl/6 and BalbC, and one strain of outbred mice MF1 (Harlan Scientific, UK). C57Bl/6 differ from BalbC mice in that BalbC mice are more ‘Th2’ prone (with emphasis in the production of antibodies) and C57Bl/6 more ‘Th1’ prone (with emphasis on the cellular immune response). Mice were housed at 21°C with a 12 h light cycle, and maintained on a diet of SDS41B food pellets (Harlan Scientific, UK) and 0.05 per cent para-aminobenzoic acid-supplemented drinking water to facilitate parasite growth [22,23]. We used two different genotypes of *Plasmodium chabaudi* that differ in virulence; AS and DK (WHO Registry of Standard Malaria Parasites, The University of Edinburgh). In adult mice, AS causes greater weight loss and anaemia than DK, and in particularly, sensitive mouse strains, such as BalbC, AS might cause some mortality [24], but see Mideo *et al*. [25]. The parasite genotypes were originally cloned from wild parasite isolates from thicket rats in the Central African Republic [26]. All infections (of dams and pups) were initiated with 0.1 ml citrate saline solution containing 10^6^ parasitized red blood cells (RBCs). Sham-(control) infected mice received only citrate saline.

### (b) Dams and breeding

For each host strain, 20 dams were infected at nine weeks of age with genotype AS, and an additional 10 dams were sham infected to generate controls. All infected dams developed malaria; confirmed by examining blood smears on 4 days post-infection. At 21 days post-infection, half of the infected dams from each strain were randomly selected and treated with 70 μg ml^−1^ pyrimethamine in their drinking water for 4 days to cure infections. As peak parasitaemia occurs 6–8 days post-infection, parasitaemia has dropped to very low levels by day 21, and the immune response has reached a plateau [27–29]. Females were not sampled for blood after drug treatment to reduce disturbance in their reproductive cycles prior to mating. However, previous work has demonstrated that a 4 day treatment of pyrimethamine clears mice from remaining parasites [16]. One month after infection, all dams were exposed to male bedding to induce oestrus and then paired with males (three dams per male, one dam, randomly chosen, for each of the treatment groups). After two weeks, males were removed and dams were put in individual cages. Dams were sampled to measure antibody levels (see below) just after weaning of their pups (to avoid disturbance during pregnancy and lactation).

Of the 30 dams for each host strain, 52 gave birth to 5.61 ± 1.18 pups on average (17 C57Bl/6 dams, 13 BalbC dams and 22 MF1 dams; table 1). Five pups died before weaning at 18 days old (see below), all from MF1 females with more than 11 pups (two from control dams, two from dams infected and drug-treated and one from an infected dam). The number of pups in the litter differed according to mouse strain (*n*_females_ = 52, *n*_pups_ = 387; ΔAIC (Akaike information criterion) to the closest model (mouse strain × maternal treatment) = 3.01, *p* = 0.0023), with MF1 mothers giving birth to significantly more pups (4.24 more ± 0.29) than the two other mouse strains that did not differ significantly (C57Bl/6 6.58 ± 0.61 and BalbC 5.61 ± 0.59 pups per dam). The ranges of litter sizes were four to nine pups for C57Bl/6 dams, 6–11 pups for BalbC and 6–13 pups for MF1. Once corrected for mouse strain, litter size did not differ according to sire identity (ΔAICc to the model with mouse strain only = 13.1, *p* = 0.30).

**Table 1.** Sample sizes according to mouse strain, maternal treatment and pup treatment.

mouse strain	maternal treatment	number of dams	mean litter size	pup treatment	total number of pups	pups sampled to survey RBC counts	pups sampled for immune assays
C57Bl/6	control	7	6.57	control	15	6	9
				DK	14	11	3
				AS	17	11	6
	infected and cured	3	7.33	control	5	3	2
				DK	7	6	1
				AS	10	6	4
	infected	7	6.28	control	9	5	4
				DK	17	12	5
				AS	18	12	6
	total	17					
BalbC	control	6	5.00	control	6	5	1
				DK	11	10	1
				AS	13	10	3
	infected and cured	3	6.00	control	4	3	1
				DK	6	5	1
				AS	8	6	2
	infected	4	6.25	control	5	3	2
				DK	9	6	3
				AS	11	6	5
	total	13					
MF1	control	7	8.71	control	16	7	9
				DK	22	13	9
				AS	23	14	9
	infected and cured	7	9.57	control	18	7	11
				DK	24	14	10
				AS	25	14	11
	infected	8	9.25	control	23	7	16
				DK	25	14	11
				AS	26	14	12
	total	22					

At 10 days old, pups were individually marked with a non-toxic marker pen and their identification was maintained throughout the experiment. Pups were weighed at 10 and 18 days old to test whether growth rate varied across strains. For pup weight at 10 days old, there was a non-significant borderline effect of maternal treatment (*n*_females_ = 52, *n*_pups_ = 382; ΔAIC to closest model (null model) = 1.85, *p* = 0.059) and no significant influence of sire identity or of host strain (*n*_females_ = 52, *n*_pups_ = 382; ΔAIC between model with host strain and maternal treatment and model with maternal treatment = 3.25, *p* = 0.095). For pup weight at 18 days, only mouse strain had a significant effect (*n*_females_ = 52, *n*_pups_ = 382; ΔAIC to closest model (strain and maternal treatment effect) = 4.71, *p* = 0.013), MF1 pups being 1.76 ± 0.48 heavier than the other mouse strains). In summary, MF1 mothers had more pups that grew faster, but no significant effect of maternal treatment on pup weight or growth was detected.

### (c) Pup infections and sampling

At approximately 18 days old (17.75 ± 0.06 days; range 17–19 days), all pups were separated from their mothers for weaning. One day after weaning, pups from each litter were divided randomly into three treatment groups: infected with AS, infected with DK or sham infected and infections were initiated for the sample sizes shown in table 1. Pups of the same family and of same sex were kept together and split in two groups when their number exceeded six individuals (this is the maximum recommended density for our cages). To minimize the amount of blood collected from each pup, individuals were either sampled for immunological assays (at weaning *n* = 151 pups) or for RBC counts and thin smears (*n* = 231 pups). The RBC/smear group was split in two cohorts; each individual was sampled every second day between days 4 and 30 post-infection (no pups died before day 4). RBC density was measured by using flow cytometry (Beckman Coulter). Thin blood smears prepared from tail blood were Giemsa stained and microscopically analysed (×1000 magnification) to calculate the proportion of parasitized RBCs.

### (d) Immunological assays

To measure parasite-specific antibodies in mothers and pups, enzyme-linked immunosorbent assays were performed. Plates (Immunosorb, NUNC) were coated with 50 μl of 1 μg ml^−1^ solution of *P. chabaudi* AS MSP1 antigen freeze–thawed crude lysate (plasmid kindly provided by J. Langhorne) in carbonate buffer. The plates were coated overnight at 4°C and the wells were blocked with 5 per cent bovine serum albumin (Sigma) in carbonate buffer for 2 h at 37°C. Each serum sample was assayed in a series of duplicate dilutions from 1 : 100 to 1 : 12800 and incubated for 1 h at 37°C. Antibody binding was detected using horseradish peroxidase-conjugated anti-murine IgG2a (Southern Biotech 1080 05) at concentration 1 : 4000 for MF1 and BalbC and 1 : 200 for C57Bl/6 (different IgG2a isotype) and ABTS peroxidase substrate (Sigma). Samples were read at 405 nm, and optical density (OD) log-transformed for normality.

### (e) Statistical analysis

Using R statistical software [30], a series of ANOVA/ANCOVA and mixed-effects models (linear mixed models and generalized additive mixed models (GAMM)) was constructed (table 2). When required, we included cage nested within dam identity as a random effect and, to take repeated measures on pups into account, pup nested within cage and dam. Survival probability was analysed using logistic regression (library lmer) with survival as a binary-dependent variable. Terms fitted in the models included pup sex, pup treatment, maternal treatment, mouse strain and their interactions. The parsimony of the null and of alternative models were assessed in an ANOVA setting using AIC or second-order AIC (AICc), when the ratio between sample size and the number of parameters tested was below 40 [31]. We report the ΔAIC or ΔAICc between the most parsimonious model and the closest model and the probability associated with the likelihood ratio test from the comparison of these models. When interactions were significant, we analysed the data for each level of relevant variables, and we report post hoc test results. Means and effects are reported ± one standard error.

**Table 2.** List of the different variables tested, and fixed and random effects used to build the models tested.

variable tested	fixed effects tested	random effects included in the model	more parsimonious model
antibody levels of dams when pups were weaned	mouse strain, maternal treatment		interaction mouse strain × maternal treatment; figure 1
antibody levels of pups at weaning (pups from infected dams)	mouse strain, maternal levels of antibody, maternal treatment, pup sex, (‘dam’ effect to test intra-litter repeatability)	cage effect nested in dam effect	maternal levels of antibody; figure 2, dam
pups survival	pup parasite genotype, maternal treatment, mouse strain, pup sex	cage effect nested in dam effect	pup parasite genotype × maternal treatment; figure 3
pups parasitemia 4 days post-infection	pup parasite genotype, maternal treatment, mouse strain, pup sex	cage effect nested in dam effect	pup parasite genotype × maternal treatment, mouse strain; figure 4
pups daily RBC counts (anaemia)	GAMM, days post-infection as smooth term, pup parasite genotype, maternal treatment, mouse strain, pup sex	cage effect nested in dam effect	pup parasite genotype × maternal treatment × mouse strain; electronic supplementary material, figure S1
lowest RBC count	pup parasite genotype, maternal treatment, mouse strain, pup sex	cage effect nested in dam effect	maternal treatment × pup parasite genotype, mouse strain; electronic supplementary material, figure S2

## 3. Results

### (a) Effect of maternal drug treatment: maternal transfer of antibodies

**Figure 1.**
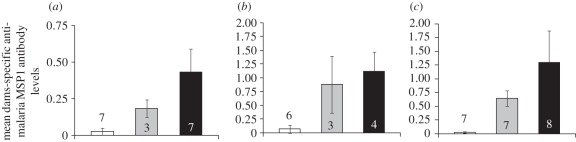
Specific anti-malaria (MSP1 antigen) antibody levels of dams at weaning of their pups, according to mouse strain: (*a*) C57Bl/6, (*b*) BalbC, (*c*) MF1 and maternal treatment. In white, control females; in grey, females exposed to malaria and drug-treated; in black, females exposed to malaria not treated. Numbers denote sample sizes, error bars 95% CI.

While the antibody levels of dams when pups were weaned were strongly affected by the interaction of maternal treatment and mouse strain (*n*_females_ = 52, ΔAICc with the closest model (mouse strain and maternal treatment) = 3.26, *p* = 0.014; figure 1), maternal treatment had a significant effect in all strains (*p* < 0.01). Control dams had significantly lower antibody levels than infected dams in all cases; in MF1 and C57Bl/6 mice, drug-treated dams had significantly lower levels than untreated infected dams; figure 1). In BalbC females, drug-treated dams were not significantly different from untreated infected dams (mean difference β = −0.23 ± 0.25, *p* = 0.54). Once corrected for maternal treatment, C57Bl/6 mice had antibody levels that were 0.45 ± 0.15 units lower than the other two strains with no significant difference between BalbC and MF1 females.

**Figure 2.**
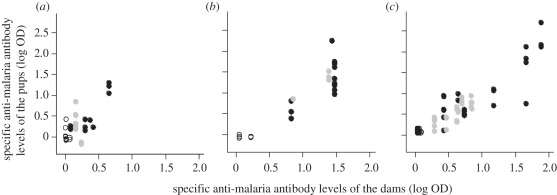
Correlation at weaning of the pups between specific anti-malaria (MSP1 antigen) antibody levels of females and their pups according to maternal treatment and mouse strains: (*a*) C57Bl/6, (*b*) BalbC, (*c*) MF1. White circles are control females, grey circles are females exposed to malaria and drug-treated, whereas black circles are females exposed to malaria not treated. Please note that because the antibody levels at weaning were not available for all the pups (see §2), not all the females are represented in this figure.

As expected, control dams and their pups had no specific anti-malaria antibodies (figure 2). In infected dams (both treated and untreated), antibody levels of the pups at weaning were strongly correlated with their mothers' levels (*n*_females_ = 26, *n*_pups_ = 86; ΔAIC to null model = 43.1, *p* < 0.0001; figure 2). The strength of the relationship was not significantly influenced by maternal treatment (*n*_females_ = 26, *n*_pups_ = 86; ΔAIC between the model with the interaction maternal treatment × pup antibody level and the model with only pup antibody level = 2.53, *p* = 0.19, β_untreated females_ = 1.05 ± 0.12; β_treated females_ = 1.01 ± 0.15). Pup antibody levels were very repeatable within each litter (model with female as fixed effect: *n*_females_ = 26, *n*_pups_ = 86; ΔAIC to null model = 136.3, *p* < 0.0001). Therefore, because not every pup could be sampled to determine antibody levels, we used maternal antibody level as the level of antibody transferred to a pup.

### (b) Effect of maternal drug treatment: survival of pups

**Figure 3.**
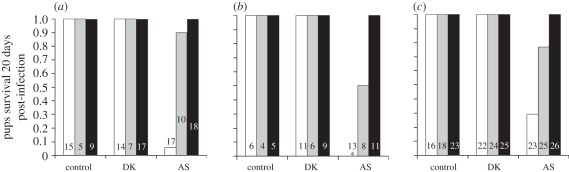
Survival of the pups according to pup treatment, maternal treatment and mouse strain: (*a*) C57Bl/6, (*b*) BalbC and (*c*) MF1. In white, pups born from control mothers; in grey, pups from mothers exposed to malaria and drug-treated; whereas in black are pups from mothers exposed to malaria not treated. Numbers denote the number of pups in each group. Asterisk denotes no BalbC pups from controls dams survived AS infection.

All pups survived infection with DK, regardless of maternal treatment. By contrast, there was variation in the mortality rate of infections with the more virulent clone, AS. The survival of pups exposed to AS was significantly different according to maternal treatment (*n*_females_ = 52, *n*_pups_ = 151; AIC = 53.4, ΔAIC to null model = 23.6, *p* < 0.0001; figure 3). Model estimates indicate that survival probability was the lowest for pups from control mothers (0.16 ± 0.084), then from drug-treated mothers (0.77 ± 0.092), and pups from non-drug-treated females were most likely to survive (0.99 ± 0.069). All pairwise comparisons were significantly different, even between the closest groups, i.e. pups from drug-treated and non-drug-treated females (*n*_females_ = 32, *n*_pups_ = 100, ΔAIC to closest model (null model) = 2.23, *p* = 0.017). No significant difference in survival was observed between mouse strains (ΔAIC between the model with the interaction mouse strain × maternal treatment and the model with only maternal treatment = 2.36, AIC = 69.9, *p* = 0.061). Furthermore, among AS-infected pups, survival probability correlated positively with maternal antibodies, even when corrected for maternal treatment (*n*_females_ = 52, *n*_pups_ = 151; ΔAIC to null model = 2.24, *p* = 0.038, once controlled for maternal treatment, survival rate increased by 0.15 ± 0.038 for each increase of one unit of antibody levels).

### (c) Effect of maternal drug treatment: levels of parasitaemia in pups

We analysed parasitaemia at 4 days post-infection (i.e. before any pups died of malaria) as this avoids problems with missing data owing to mortality of the most severe infections. For pups exposed to AS, parasitaemia (proportion of infected RBC) was significantly influenced by maternal treatment and mouse strain (*n*_pups_ = 44; ΔAICc to closest model (model with maternal treatment only) = 2.96, *p* = 0.039). Once mouse strain was controlled for, all the maternal treatments differed from each other (estimated mean parasitaemia: pups from control females 0.31 ± 0.046, from females infected and drug-treated 0.10 ± 0.045 and from females infected and not drug-treated 0.0081 ± 0.033). For pups exposed to DK, parasitaemia significantly differed according to maternal treatment and mouse strain (*n*_pups_ = 47, ΔAICc to closest model (model with maternal treatment × mouse strain interaction) = 5.26, *p* = 0.0033). However, once corrected for strain, parasitaemia of pup infections from control and drug-treated females differed from infected females but not from each other (estimated mean parasitaemia: pups from control females 0.11 ± 0.023, from females infected and drug-treated 0.089 ± 0.017 and from females infected and not drug-treated 0.044 ± 0.013).

**Figure 4.**
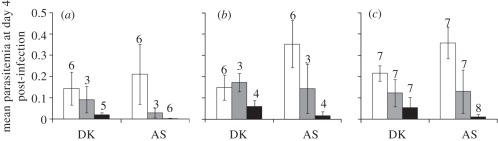
Mean parasitaemia of pups 4 days post-infection, according to pup treatment (infected with AS or DK malaria strain), to mouse strain: (*a*) C57Bl/6, (*b*) BalbC, (*c*) MF1 and maternal treatment. In white, pups from control females; in grey, from females exposed to malaria and drug-treated; whereas in black, from females exposed to malaria not treated. Numbers denote sample sizes, error bars 95% CI. NB: all the control pups (sham infected) had no parasites (0% parasitaemia).

The overall analysis (on both AS- and DK-infected pups) indicated that the most parsimonious model involves an interaction between pup treatment × maternal treatment and mouse strain (*n*_pups_ = 139; ΔAICc to closest model (model with maternal treatment × pup treatment interaction) = 6.48, *p* = 0.0051; figure 4). This demonstrates that the patterns described above are comparable for each mouse strain, but that pups from C57Bl/6 had parasitaemia lower than the two other strains (mean difference between C57Bl/6 pups and other pups, β = −0.042 ± 0.017, *p* = 0.0152). Finally, in accordance with the higher virulence of AS, post hoc tests indicated that, once maternal treatment is controlled for, parasitaemia was 0.039 ± 0.012 higher for AS compared with DK-infected pups (*p* = 0.026), which were 0.12 ± 0.021 higher than controls (*p* < 0.0001).

### (d) Effect of maternal drug treatment: anaemia of the pups

The anaemia experienced by pups, measured as RBC counts in relation to days post-infection, depends on the interaction between pup treatment, maternal treatment and the host strain (GAMM models, *n*_pups_ = 231, ΔAIC to closest model (interaction maternal treatment and pup treatment) = 58.1, *p* < 0.0001; electronic supplementary material, figure S1). To describe this triple interaction in more detail, we focus on the lowest mean RBC counts (electronic supplementary material, figure S2). The day of lowest RBC count varied between 4 and 10 days post-infection across treatment groups, reflecting different infection dynamics across maternal treatments (see GAMM models in the electronic supplementary material). Once corrected for mouse strain (see below), pups from infected dams had significantly higher minimum RBC counts (for DK-infected pups *n*_pups_ = 38, β = −1.98 ± 0.42, *p* < 0.0001; for AS-infected pups *n*_pups_ = 39, β = −2.89 ± 0.51, *p* < 0.0001), but pups from dams not infected or infected and drug cured had similar minimal RBC counts (for DK-infected pups *n*_pups_ = 32, β = −0.51 ± 0.45, *p* = 0.69; for AS-infected pups *n*_pups_ = 22, β = −0.81 ± 0.53, *p* = 0.33).

As observed for parasitaemia, lowest mean RBC counts were affected by the interaction between pup treatment × maternal treatment and by a simple effect of mouse strain (*n*_pups_ = 123; ΔAICc to closest model (model with maternal treatment × pup treatment interaction) = 2.73, *p* = 0.046). This means that the patterns that we have described were comparable for each mouse strain, except that pups from C57Bl/6 had RBC counts slightly lower than the two other strains (mean difference between C57Bl/6 pups and other pups β = −0.49 ± 0.21, *p* = 0.044).

## 4. Discussion

In this study, we investigated the role that within-host infection dynamics play in the fitness consequences of maternally transferred immunity. Using rodent malaria as a model system, we manipulated maternal infection and compared the levels of antibody transferred to offspring, and the resulting morbidity and mortality of experimental infections in pups. Our results clearly show, across three different host strains and two different parasite genotypes, that pups born to dams previously challenged with malaria infection experience lower parasitaemia and are more likely to survive than pups from naïve dams. Moreover, our results also demonstrate that maternal transfer of protection is substantially decreased when drugs were administered at three weeks post-infection to cure dams. Drug treatment against malaria reduced the amount of antibody transferred, resulting in higher parasitaemia infections in pups and reduced survival probability compared with pups from infected dams that were not treated.

### (a) Maternal immunity and protection of the pups

Our results indicate a correlation between the levels of maternally transferred immunity and the protection conferred to the pups. Maternal exposure to malaria had a positive effect on the survival of pups to infection. Moreover, our results suggest that the degree of protection conferred to the pups, measured in terms of enhanced survival, is correlated with the amount of antibodies received (even after controlling for maternal treatment). Several studies [32–36] (but see Williams *et al*. [37]) have shown that an immune challenge might affect maternal condition, feeding rate and consequently reproductive success of breeding animals. The cost of mounting an immune response and coping with malaria infection may indeed affect post parental care [38] and prenatal maternal effects [39]. However, in our experiment, while maternal exposure to malaria affected pregnancy rates, it did not decrease the weight or growth rate of the pups, and had a positive effect on the survival of challenged pups. Experimental manipulations of the amount of antibodies transferred to the pups are now required to test the link we observe between maternally transferred antibodies and offspring survival [40,41]. An alternative hypothesis to explain the transfer of protection from mothers exposed to malaria is that maternally transferred immune complexes ‘prime’ the immune response of pups. May *et al.* [42] have recently observed, in humans, an antibody-dependent trans-placental transfer of an antigen in the form of immune complexes that could prime the immune system of the young (but see Soulard *et al*. [43] and Malhotra *et al*. [44]). Disentangling pre- and post-natal effects of maternally transferred immunity and priming of the offspring immune system is challenging, but rodent malaria models offer a number of useful tools. This includes the experimental manipulation of maternal infection (as we have demonstrated here) [45], cross-fostering manipulations [19,46,47], the use of different mouse strains that vary in the characteristics of their immune responses [15] and cross-breeding of mouse strains to distinguish between maternally transferred antibodies and antibodies produced by pups [21].

### (b) Effect of maternal drug treatment

The dynamics of infection can have major effects on the morbidity and mortality risk of infection in adults [8], but the consequences for offspring remain largely unknown because it has rarely been manipulated experimentally [4,8]. By shortening the duration of infection experienced by adults, drug treatments can negatively affect the breadth of the immune response by reducing the range and the amount of antigens expressed by the parasite [11]. Alternatively, it is possible that drug treatment can render different parasite proteins accessible to the host immune system, inducing quantitative and qualitative changes in antigen recognition, which could include antigens not exposed by parasites under normal conditions [12,13]. Testing these hypotheses requires data on the affinity and avidity of maternal antibodies to parasite antigens exposed during infections in offspring. It, therefore, remains unclear whether, in adults, a protective response is induced after treatment against parasites, how it differs from acquired immunity to ‘natural’ infection [48,49] and how this affects levels of protection conferred to offspring [50].

Studies performed in natural conditions suggest that exposure to parasites is correlated with the amount of antibodies transferred [51–53] to offspring and that the inter-annual pattern of exposure to parasites affects the transfer of a protective repertoire of antibodies [54,55]. However, experimental manipulation of the dynamics of maternal exposure is needed to test the consequences of maternal exposure for the transfer of protection. Our results show that shorter maternal infections induce less protection for offspring than full infections. Moreover, drug treatment—even towards the end of the acute phase of infection—curtails infections enough to reduce maternally transferred protection. More broadly, our results highlight that treating maternal infection as a presence/absence factor is not sufficient to evaluate the adaptive value of this maternal effect in natural conditions [3]. Future studies should focus on testing how the length of infection interacts with other characteristics of maternal infection (such as timing of the infection, infectious dose and infection genetic diversity) to shape the amount of antibodies transferred and the level of protection conferred to offspring. These experiments could also distinguish two alternative hypotheses; shortened maternal exposure to parasites enhances maternal condition and consequently offspring fitness, or it decreases maternally transferred immunity and fitness of the young if it is exposed to parasites.

### (c) Effect of mouse strain

Inbred mouse strains differ in key characteristic of the immune response. For example, BalbC mice are more ‘Th2’ prone (and therefore emphasizing the production of antibodies) and C57Bl/6 more ‘Th1’ prone (and therefore emphasizing the cellular immune response). Immunity to blood-stage malaria infections involves both antibody-dependent and independent mechanisms, and T-cells play a crucial role in the induction and maintenance of this immunity [56]. After an initial Th1 response required to control the acute infection [57–59], mice develop a Th2 response with subsequent B-cell activation, producing antibodies [60]. Despite the differences in the Th1- and Th2-oriented immune responses of BalbC and C57Bl/6 strains, Helmby *et al.* [61] have shown that both strains develop anti-malarial IgG at a similar rate and magnitude after repeated exposures to malaria [61]. As maternal immunity relies on the production of antibodies, we tested if the difference in the initial orientation of maternal immune responses of C57Bl/6 and BalbC strains might affect the protection transferred to the pups. Our results indicate that the protection conferred to the pups is affected in the same way by different maternal treatments across C57Bl/6, BalbC and an outbred strain (MF1). However, while qualitative patterns matched, C57Bl/6 pups had overall lower parasitaemia, slightly lower RBC counts (even for control pups) and the C57Bl/6 dams produced and transferred less antibodies to their pups. This latter result can potentially be explained by a Th1-oriented immune response of this mouse strain but lower RBC counts, observed even for control pups, are not usually described for this mouse strain [62] even though only data on adults are available. More detailed data on the dynamics of infections in pups, coupled with mathematical modelling will help reveal the mechanisms underpinning strain variation in infection dynamics.

### (d) Conclusions

Our results show that anti-parasitic treatments are powerful tools to manipulate the dynamics of maternal infection and study the impact on maternally transferred protection to offspring. We observed the same pattern—that drug treatment of dams reduces maternally transferred immunity to pups—across three different mouse strains and after exposing pups to different parasite genotypes. Explaining the impact of infection dynamics and genetic background on the protection conferred by the mother is a key factor underpinning inter-individual [3] and interspecific [63] variability of maternally transferred immunity observed in natural conditions. More broadly, understanding these relationships may be important for the design of public health policies. Given the inconclusive evidence on the effect of maternal treatment on human infants' survival of malaria infection [50] and immune response [44], our results highlight the need to investigate this issue. The effect of treatment on the acquisition of immunity is particularly important for individuals in endemic areas where re-infection is likely to occur and treatment against parasitic disease is widely used. In these endemic areas, infants are likely to be exposed to the same diseases as their parents and at an age when their immune system is not fully developed. They consequently rely on the repertoire of antibodies transferred by their mothers to confer passive protection. However, as maternal treatment against parasites substantially decreases the amount of antibodies transferred, resulting in higher parasitaemia infections in pups and reducing their survival probability, we reveal potential trade-off between maximizing the health of mothers and offspring.
